# Phenological Analysis of Grasses (Poaceae) in Comparison with Aerobiological Data in Moscow (Russia)

**DOI:** 10.3390/plants13172384

**Published:** 2024-08-27

**Authors:** Elena E. Severova, Vera S. Karaseva, Yulia M. Selezneva, Svetlana S. Polevova

**Affiliations:** 1 Faculty of Biology, Shenzhen MSU-BIT University, Shenzhen 518172, China; 2 Biological Faculty, Moscow State University, 119991 Moscow, Russia; svetlanapolevova@mail.ru; 3 Institute of Natural Science, S.A. Esenin Ryazan’ State University, 390000 Ryazan, Russia; v.karaseva@365.rsu.edu.ru (V.S.K.); posevina_julia@mail.ru (Y.M.S.)

**Keywords:** Poaceae, phenological index, pollen concentration, Russia

## Abstract

Grasses (Poaceae) produce large amounts of pollen and are among the main causes of pollinosis worldwide. Despite their morphological similarity, pollen grains of different grass species may have different allergenicities. Therefore, quantification of the roles of individual species in airborne pollen is an important task. There are very few studies on this topic, and none of them have been conducted in a temperate continental climate. Our study was carried out for three years (2020–2022) in the urban territory of Moscow (Russia) and aimed to understand what grass species contribute the most to the total pollen load of the atmosphere. The comparison of aerobiological and phenological data was based on calculating the phenological index, which is a combination of phenological parameters, pollen productivity of individual species, and their abundance. Our data showed that the decomposition of pollination curves based on the phenological index was sometimes very efficient but not always possible in temperate continental climates. The main reasons for disagreement between aerobiological and phenological data were weather conditions and lawn mowing. Not all grasses were equally important as sources of allergenic pollen. The greatest contribution to the pollen load at the beginning of the season in Moscow was made by *Dactylis glomerata*, and to a lesser extent by *Phleum pratense* and *Festuca pratensis*. These are the most common species, which are widespread throughout Europe. The contribution of minor components is insignificant.

## 1. Introduction

Grasses (Poaceae) form the second-largest monocot family, comprising more than 11,000 species [1,2]. The significance of this group lies not only in the number of species and their wide distribution but also in their use as cereal and pasture plants since the Neolithic [3,4,5]. Most grasses are wind-pollinated [1,6], with the exceptions of some cleistogamous [7] and a few entomophilous species [8]. They produce a large amount of pollen [9,10,11,12,13,14], which is among the main causes of pollinosis worldwide [15,16]. According to structural and biological properties, eleven groups of allergens have been identified in grass pollen. The groups differ in the degree of exposure to sufferers from pollinosis; groups 1 and 5 are responsible for most cases of grass allergy (major allergens), causing sensitization in 90% and 65–85% of patients, respectively [17,18]. It has been shown that the prevalence of sensitization to grass pollen is significantly higher than to pollen of other plants [19,20]. In this connection, aerobiological monitoring is of primary importance for the interpretation of allergy data and therapy planning. Routine monitoring is usually carried out by volumetric pollen and spore traps of Hirst-type [21], which implies pollen identification under a light microscope. Poaceae is a stenopalynous family; pollen grains of all species belong to a single morphological type, so it is impossible to distinguish pollen of different genera using this method [22]. Pollen curves and calendars obtained by standard aerobiological monitoring usually present grasses as one group. Determination of the flowering time of grass species can be important for allergy suffers as it is assumed that pollen of different species may have unequal allergenicity [23,24]. This may be reflected in the different responses of patients to the same concentration of grass pollen during the pollen season, as noted in de Weger et al. [25]. Despite significant cross-reactivity [26,27], the allergens of various grasses differ. This may be related to different protein isoforms, alternative splicing, or post-transcriptional modifications [18]. Accurate identification of the source of allergenic proteins is crucial in modern allergen-specific immunotherapy. Detailing the grass pollination curve up to the genus or species level is possible either based on phenological observations [28,29] or by metagenomic methods [30,31,32,33,34,35,36,37,38].

Phenological analysis has already been used to interpret aerobiological data for both arboreal [39,40,41] and herbaceous [28,29,42,43,44,45] taxa. A combination of aerobiological and phenological data could help to correlate symptoms with the pollination of specific taxa and implement immunospecific therapy. However, phenological studies in aerobiology are not common as they need specific botanical knowledge and a long period of observation. A few studies on grass phenology and aerobiology were carried out in Italy [28,29], Spain [42,43,44], and Austria [45].

Our study was focused on the urban territory of Moscow (Russia) and aimed to understand what grass species contribute the most to the total pollen load of the atmosphere. Moscow is one of the largest metropolises in Europe, with the highest population and a high level of pollinosis [46]. The vegetation and climate of Moscow are significantly different from those regions where studies on the phenology and pollen emission of various grass species were previously conducted. Therefore, our study may have both theoretical and practical significance.

## 2. Results

**Figure 1:**
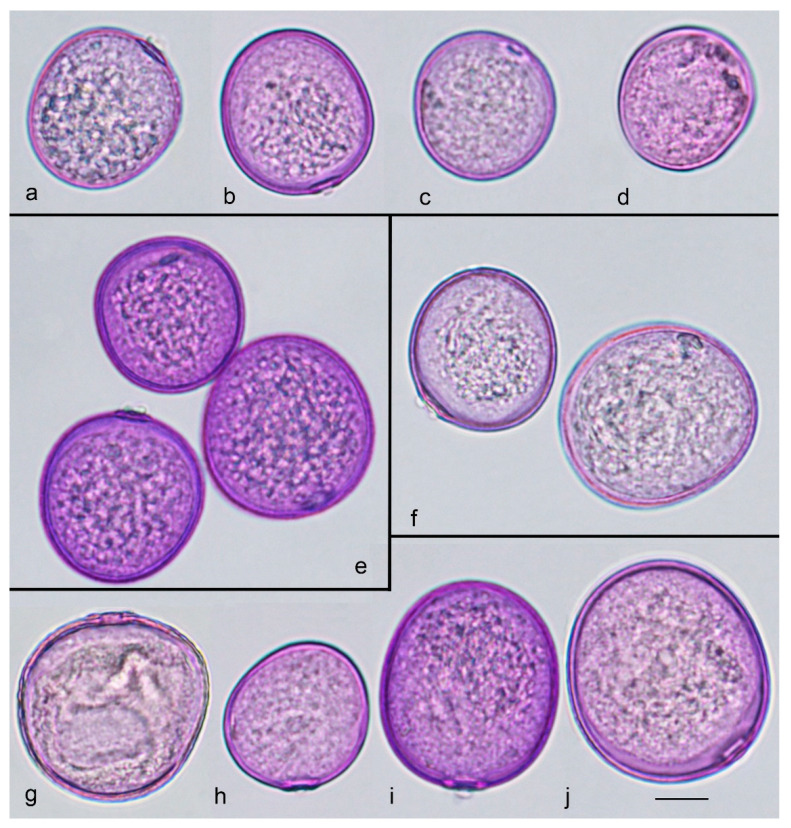
Pollen of Poaceae species under a light microscope, fuchsine-stained: (**a**) *Arrhenatherum elatius*, (**b**) *Festuca pratensis*, (**c**) *Poa pratensis*, (**d**) *Poa trivialis*, (**e**) *Dactylis glomerata*, (**f**) *Phleum pratense*, (**g**) *Bromopsis inermis*, (**h**) *Calamagrostis epigeios*, (**i**) *Lolium perenne*, and (**j**) *Elymus repens*. Scale bar, 10 µm.

The following ten grass species were identified on sample plots: *Arrhenatherum elatius* (L.) P. Beauv. ex J. and C. Presl. (MW 1072498), *Bromopsis inermis* Leyss. (MW 1072504), *Calamagrostis epigeios* (L.) Roth (MW 1072496), *Dactylis glomerata* L. (MW 1072497), *Elymus repens* (L.) Gould (MW 1072493), *Festuca pratensis* Huds. (MW 1072495), *Lolium perenne* L. (MW 1072494), *Phleum pratense* L. (MW 1072502), *Poa pratensis* L. (MW 1072499), and *Poa trivialis* L. Pollen of all species was studied and photographed under a light microscope (Figure 1). A biostatistical analysis of the average diameter of the pollen grains could discriminate some species (for data and analysis, see [14]), but such measurements would not be very reliable because of the high intra-group variability.

**Figure 2:**
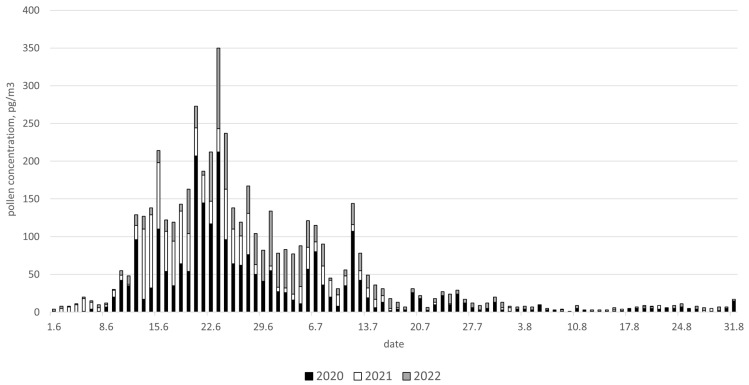
Dynamics of grass pollen concentration, 2020–2022; a stalked chart.

In all studied years, the grass pollen season started at the beginning of June and lasted until the end of July. The intensity of pollination differed considerably among seasons. The highest one was in 2020; the seasonal pollen integral (SPIn) in 2020 was almost two times higher compared with 2021 and 2022 (Figure 2).

**Figure 3:**
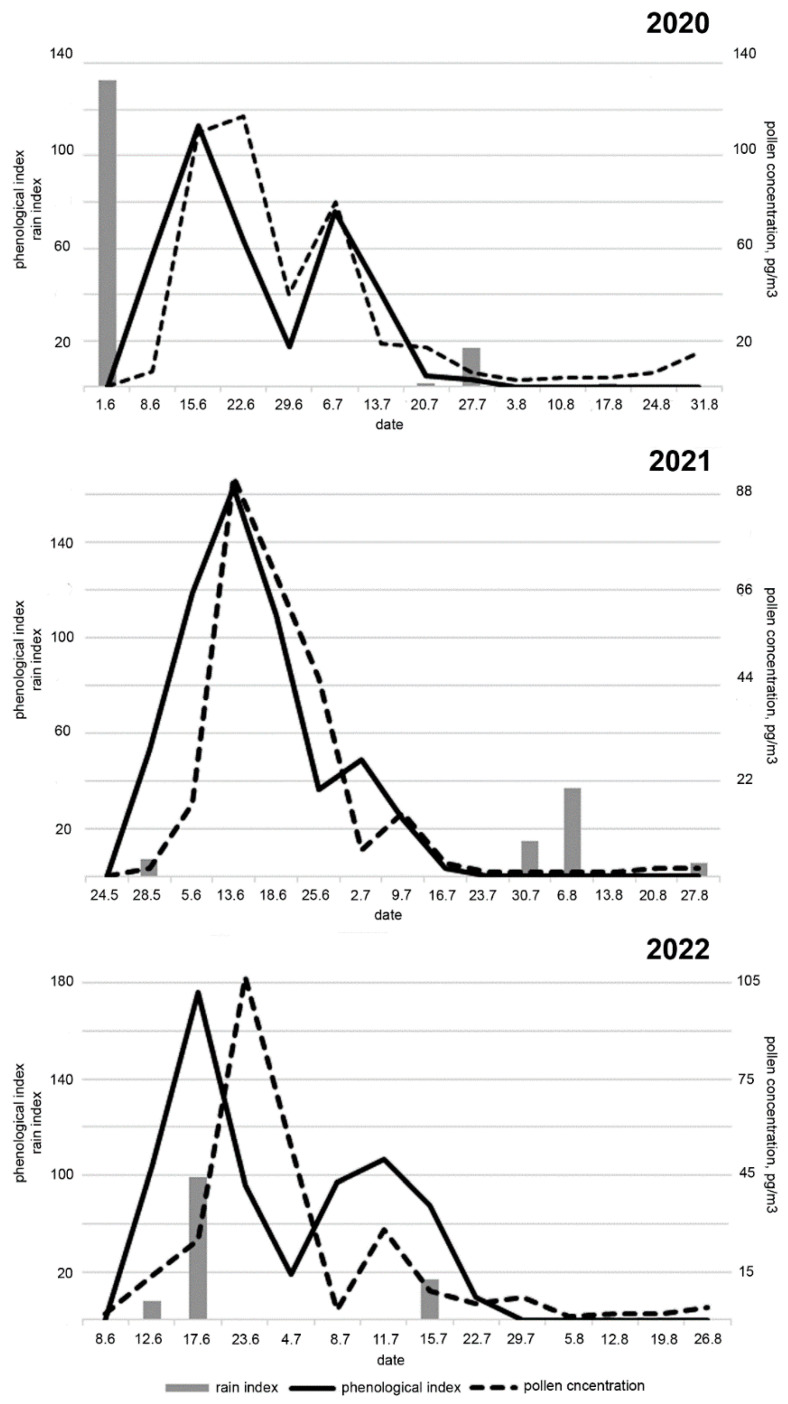
Comparison between phenological index and grass pollen concentration, 2020–2022.

The phenological index (PHI) and grass pollen concentration on the same day had high correlation coefficients in 2020 and 2021 (r = 0.83, *p* = 0.0002 and r = 0.82, *p* = 0.00016, respectively), but it was low and insignificant in 2022 (r = 0.34, *p* = 0.23) (Figure 3).

**Figure 4:**
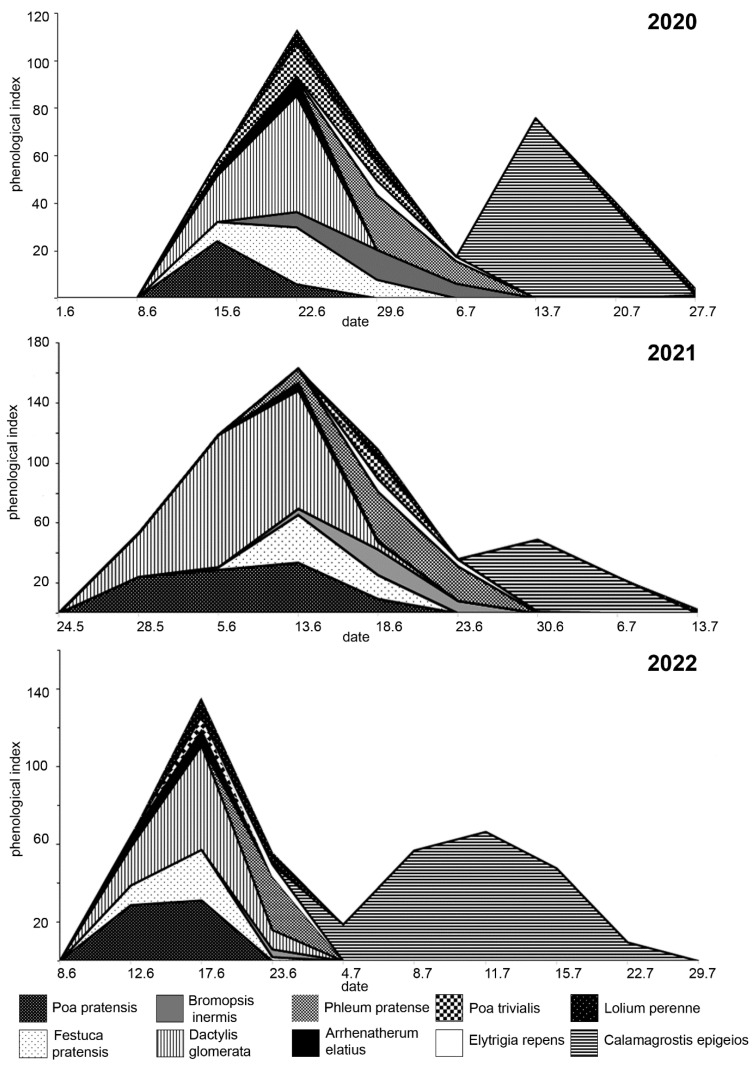
Decomposition of the cumulative PHI curve into PHI curves for individual taxa.

The decomposition of the PHI curves was performed based on individual (single-species) phenological indexes. Figure 4 shows the contribution of each taxon to the summed curve of PHI. Taxa whose individual indexes exceed 10% of the total are considered the main sources of grass pollen. A list of these taxa is presented in Table 1.

The curve of PHI had two distinct peaks, the first one took place in the second half of June, and the second one was in the middle of July. *Dactylis glomerata* and *Calamagrostis epigeios* were the main contributors to the total PHI curves in all seasons. Pollen of *Dactylis glomerata* dominated in the first peak, and *Calamagrostis epigeios* dominated the second one. Apart from these two species, a significant contribution to the cumulative PHI curve was made by *Festuca pratensis* (in 2020 and 2022), *Poa pratensis* (in 2021 and 2022), and *Phleum pratense* (in 2021).

## 3. Discussion

Significant fluctuations in the SPIn of grasses have been reported earlier both in our study [47] and in other works [48,49,50,51,52,53]. Several studies have shown that SPIn can be influenced by pre-seasonal precipitation [40,54,55,56]. In regions with a continental temperate climate, precipitation is not a limiting factor as it is in more southern and drier regions [47,57]. Apparently, land use is much more significant in urban territories than meteorological differences between seasons. Modification of land use and mowing regimes could change the abundance of Poaceae species and the amount of produced pollen considerably [48,58]. If the mowing regime is chaotic and not consistent with grass phenology over the seasons, fluctuations in SPIn would be unpredictable. The impact of lawn mowing on the SPIn is visible in our study. In 2020, intensive lawn mowing began only at the end of June because of the strict COVID-19 lockdown. By that time, most of the grasses had already finished pollination. As a result, the SPIn of grasses in 2020 was the highest in a thirty-year observation period and twice as high as SPIn in 2021 and 2022 [47].

The study by Ghitarrini et al. [29] demonstrated that the dynamics of PHI are in good agreement with aerobiological observations, and thus, the phenological index could be a good estimation of the contribution of various grass species to the airborne grass pollen load.

In our work, good correlations were shown only for two seasons (2020 and 2021); in 2022, the correlation between these two parameters was weak. We consider rainfall to be the main reason for this disagreement. The maximum values of PHI are usually observed in the second decade of June. In 2020 and 2021, during this period, the weather was mostly dry (three days of the decade with rain), and in 2022, this period was rainy (8 days with rain) (Figure 3). Daily rains washed pollen out of the atmosphere. After the rains stopped on the 22nd of June, the concentration of pollen in the air increased significantly. The pollination curve turned out to be shifted relative to the PHI curve by one step (4 days); the correlation between the curves with this lag was 0.85 (*p* = 0.0001).

As shown earlier [59], a pollen trap installed 10–12 m above ground level on the Central Russian plain can reflect pollination in an area with a radius of 50 km. Phenological observations carried out at one point, even on several sample plots, could not reflect flowering in such a large area. This can be one of the reasons for disagreement between phenological and aerobiological data.

The shift in the pollen concentration peak relative to the PHI increase can also be explained by the “metropolis effect”. According to Dmitriev and Bessonov [60], the phenological stages of plants in a city are significantly ahead (up to ten days) of the same stages outside the metropolis. This is associated with a higher average daily temperature because of difficult heat removal and a huge amount of stone and asphalt. Thus, some delay in the pollen curve relative to PHI may reflect pollination not in the city, but outside it. However, the greatest influence on grass pollen concentration in the city is lawn mowing. As a rule, phenological observations are carried out on undisturbed sample plots, which makes it possible to trace the phenology. A city pollen trap reflects pollination in urban areas that are actively mowed. Intensive lawn mowing leads not only to a change in SPIn but also to a disagreement between phenological and aerobiological observations.

The individual PHI of only three to four species made an essential contribution to the cumulative PHI. Of these, *Dactylis glomerata* was the most important and made the maximum contribution to the pollen load in June. This species is noted as one of the main sources of grass pollen in several regions [10,13,29,45]. The second most important species was *Calamagrostis epigeios*, whose flowering is associated with an increase in pollen concentration in the middle of July. Despite the lower production of pollen per anther compared with other perennial grasses, the total pollen production of this species is very high because of the large number of spikelets in the inflorescence [14]. We were unable to find information on the allergenicity of the pollen of *C. epigeios*, but the pollen of related species *C. canadensis* and *C. rubescences* are classified as mild allergenic (https://www.pollenlibrary.com (accessed on 01 July 2024)).

*Poa pratensis* was found at almost all sampling plots, and its flowering made a significant load on the cumulative PHI curve; however, its pollen is moderately allergenic (https://www.pollenlibrary.com (accessed on 01 July 2024))) and may not have a significant effect on the development of hay fever symptoms. In contrast, the pollen grains of *Festuca pratensis* and *Phleum pratense* are known as severe-causing factors [16,61,62]. Both species are widespread in the city, and species of *Festuca* are used in lawn mixtures; thus, the pollen of these species, along with *Dactylis*, can be an elicitor of June pollinosis.

## 4. Material and Methods

This study was performed in 2020–2022 in the city of Moscow (Russia) located in the middle of the East European Plain, 156 m above sea level. The city covers an area of 2562 km^2^ and has about 13 million inhabitants [46]. The climate in Moscow is continental temperate, the coolest month is usually January (−6.2 °C), and the warmest one is July (19.7 °C) (average for the years 1981–2010). The mean total annual rainfall is 684 mm, and the average period with snow cover is 134 days [60].

### 4.1. Aerobiological Data

Daily average concentrations of grass pollen were obtained according to the standard aerobiological method [63]. Pollen sampling was performed using a Hirst-type volumetric sampler [21] located on an open roof of a meteorological station, 10 m above ground level (55°42′ N, 37°32′ E). The meteorological station is located within the Moscow State University campus near a nature reserve territory and close to the University Botanical Garden. The surrounding vegetation is dominated by *Fraxinus* L., *Acer* L., *Quercus* L., *Betula* L., *Malus* P. Mill., and *Populus* L.; *Ulmus* L. and *Picea* A. Dietr. are commonly used in urban greening. Within a radius of 1.5 km around the trap location, grass lawns occupy about 11.5%, waste ground 3%, and the area of tree planting about 10% [47]. Pollen samples were analyzed under a Nikon Eclipse Ci light microscope (Nikon GMBH, Dusseldorf, Germany) by 12 equidistant transverse strips every 2 h. Results were expressed as a number of pollen grains per cubic meter of air (pg/m^3^).

### 4.2. Meteorological Data

Meteorological data were provided by the Meteorological Station of Moscow State University located in the immediate vicinity of the monitoring site. We used daily total rainfall and rainfall duration per day. For comparison with phenological and aerobiological data, we calculated a rain index, which is a product of precipitation intensity and rainfall duration.

### 4.3. Phenological Data

**Figure 5:**
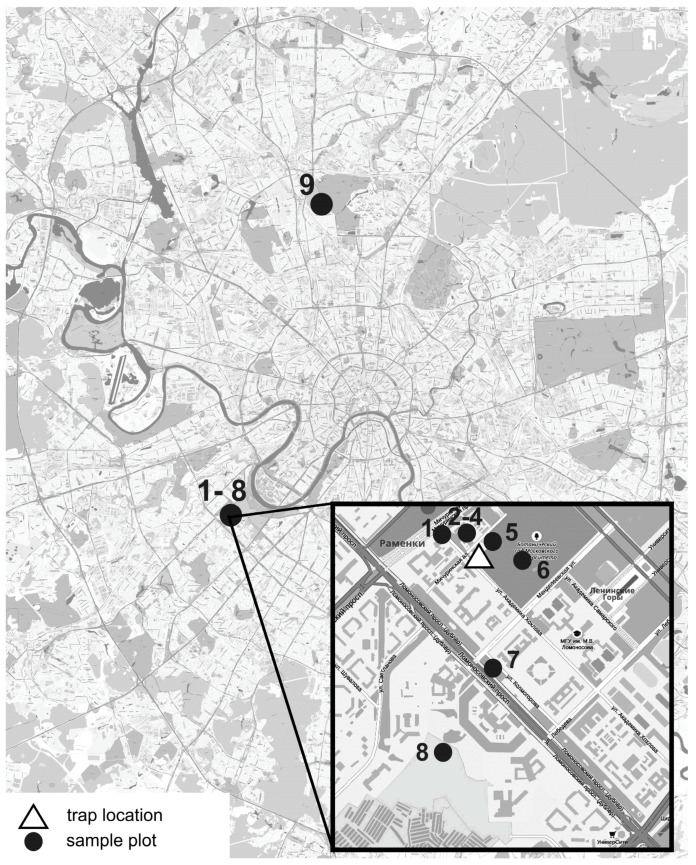
Survey area, 1–9—the numbers of sample plots.

For phenological observations and calculation of the phenological index (PHI) we used the modified method proposed by Ghitarrini et al. [29]. For phenological observations, 9 sampling plots were selected in the city, most of them in close vicinity to the pollen trap location (Figure 5). In order to check the difference in grass phenology, one sample plot was located 14.5 km north of the pollen trap. The sampling plots represented different urban green spaces, mostly grass lawns and waste ground; a list of sampling plots is presented in Table 2. Phenological stages were determined according to the BBCH scale [64]. The observations were carried out over 3 months (the end of May–the end of August), and each sampling plot was visited once every 4–7 days. For each grass species, at least 25 individual plants were checked. The observed species are listed in Table 2.

According to Ghitarrini et al. [29], PHI was calculated weekly for each species on each sample plot. The phenological index is a combination of the following three parameters: phenological stage, species abundance, and pollen productivity. For the calculations, BBCH scale stages were scored as follows: stage 63–65 (full flowering: all stamens extruded, full pollen emission) received score 2, stages 60–62 (stamens partially extruded) and 66–68 (part of stamens withered) received score 1, and all other stages received score zero [29]. It is well-known that pollen production per inflorescence differs significantly between annuals and perennials. The greater pollen production of perennial plants can be interpreted as a tendency to guarantee the cross-fertilization of species with self-incompatibility, which is typical for many perennial grasses [7,65]. As all observed species on the sample plots were perennial, we used direct calculations of their pollen production carried out at the same location; the data was published earlier [14]. To estimate the abundance of grass species, we used two categories—non-prevailing (score 1) and prevailing (score 2) following [29].

The weekly PHIs of each species were summed over all sample plots to evaluate the contribution of every species to the total pollen load. Summed weekly PHI of all species was compared with aerobiological data by correlation analysis. Data analysis was performed in R 4.0.5.

## 5. Conclusions

Decomposition of pollination curves based on phenological observations is sometimes very efficient, but not always possible. The disagreement between aerobiological and phenological data may be due to weather conditions, intensive lawn mowing, the “metropolitan effect”, or the transport of pollen from other regions. An analysis of PHI dynamics showed that not all grasses were equally important as sources of allergenic pollen. In temperate continental climates in the urban environment, the greatest contribution to the pollen load at the beginning of the season was made by *Dactylis glomerata*, and to a lesser extent by *Phleum pratense* and *Festuca pratensis*. The flowering of *Calamagrostis epigeios* was associated with an increase in pollen concentration in the second half of the season, but this second peak was of lesser importance because of the mild allergenicity of *Calamagrostis* pollen. More accurate identification of grass pollen in the air requires other research methods and can be implemented using molecular genetic approaches.

## Figures and Tables

**Table 1:** The list of grass species with the biggest contribution to the cumulative PHI.

Taxon	Contribution to the Cumulative Phenological Index, %		
	2020	2021	2022
*Festuca pratensis*	11		12
*Dactylis glomerata*	18	33	24
*Calamagrostis epigeios*	30	12	28
*Poa pratensis*		12	18
*Phleum pratense*		11	

**Table 2:** Description of sample plots.

Sample Plot	Type of Vegetation	Taxa of Grasses Identified in the Phenological Survey (Prevalent Species in Bold)	Distance from the Trap Location
1	Lawn	***Dactylis glomerata*** *Festuca pratensis* *Lolium perenne* *Phleum pratense* *Poa pratensis*	160 m
2	Waste ground	*Calamagrostis epigeios* ***Dactylis glomerata*** *Elymus repens* *Festuca pratensis* *Phleum pratense* ***Poa pratensis*** *Poa trivialis*	60 m
3	Waste ground	*Calamagrostis epigeios* ***Elymus repens*** *Poa pratensis* *Poa trivialis*	80 m
4	Lawn	*Festuca pratensis* *Lolium perenne* ***Phleum pratense*** *Poa pratensis*	36 m
5	Lawn	***Arrhenatherum elatius*** *Dactylis glomerata* *Elymus repens* *Festuca pratensis* *Lolium perenne* *Phleum pratense* *Poa pratensis*	10 m
6	Unmown area in the Moscow University Botanical Garden	*Arrhenatherum elatius* ***Bromopsis inermis*** *Calamagrostis epigeios* *Dactylis glomerata* *Elymus repens* ***Festuca pratensis*** *Lolium perenne* *Phleum pratense* *Poa pratensis* *Poa trivialis*	200 m
7	Lawn	***Arrhenatherum elatius*** *Dactylis glomerata* *Festuca pratensis* *Poa pratensis* *Poa trivialis*	530 m
8	Waste ground	*Bromopsis inermis* ***Calamagrostis epigeios*** *Dactylis glomerata* *Elymus repens* ***Festuca pratensis*** ***Poa pratensis*** *Poa trivialis*	890 m
9	Lawn	*Bromopsis inermis* ***Dactylis glomerata*** *Elymus repens* *Festuca pratensis* ***Lolium perenne*** ***Phleum pratense*** *Poa pratensis*	14.5 km

## Data Availability

Data is contained within the article, further inquiries can be directed to the corresponding author. High-quality images of the studied species are available through the data portal of the Herbarium of Moscow State Univeristy (https://plant.depo.msu.ru/open/public/search?collection=MW, accessed on 5 July 2024).
